# Robust homology-directed repair within mouse mammary tissue is not specifically affected by *Brca2* mutation

**DOI:** 10.1038/ncomms13241

**Published:** 2016-10-25

**Authors:** Elizabeth M. Kass, Pei Xin Lim, Hildur R. Helgadottir, Mary Ellen Moynahan, Maria Jasin

**Affiliations:** 1 Developmental Biology Program, Memorial Sloan Kettering Cancer Center, 1275 York Avenue, New York, New York 10065, USA; 2 Department of Medicine, Memorial Sloan Kettering Cancer Center, 1275 York Avenue, New York, New York 10065, USA

## Abstract

The mammary gland undergoes significant proliferative stages after birth, but little is known about how the developmental changes impact DNA double-strand break (DSB) repair. Mutations in multiple genes involved in homology-directed repair (HDR), considered a particularly accurate pathway for repairing DSBs, are linked to breast cancer susceptibility, including *BRCA2*. Using reporter mice that express an inducible endonuclease, we find that HDR is particularly robust in mammary tissue during puberty and pregnancy, accounting for 34–40% of detected repair events, more than in other tissues examined. *Brca2* hypomorphic mutation leads to HDR defects in mammary epithelium during puberty and pregnancy, including in different epithelial lineages. Notably, a similar dependence on *Brca2* is observed in other proliferative tissues, including small intestine epithelium. Our results suggest that the greater reliance on HDR in the proliferating mammary gland, rather than a specific dependence on BRCA2, may increase its susceptibility to tumorigenesis incurred by *BRCA2* mutation.

## Abstract (web summary)

Mutations in homology-directed repair genes like *BRCA2* are linked to breast cancer susceptibility. Here the authors generate mice with an inducible DNA break-reporter system and see high levels of homology-directed repair in proliferative mammary tissue and a general reliance on BRCA2 in various tissues.

DNA double-strand breaks (DSBs) in mammalian cells are repaired by two major pathways, homology-directed repair (HDR), and non-homologous end joining (NHEJ)^1^. HDR is considered the more precise of the pathways because it usually involves repair from the identical sister chromatid^2^, whereas NHEJ can be prone to errors. NHEJ is often thought to be the predominant pathway for repair^3^,^4^, especially in the animal where most somatic cells are not cycling. However, quantitative *in vivo* measurements in tissues to accurately assess the contribution of each pathway to DSB repair have been lacking.

In their capacity as genomic caretakers, many HDR genes are breast tumour suppressors^5^,^6^, including *BRCA1* and *BRCA2*, suggesting the importance of this DNA repair pathway for tissues or tissue compartments that proliferate postnatally and may thus be particularly at oncogenic risk when breaches occur to this pathway. The mammary gland undergoes significant postnatal development during puberty and pregnancy^7^. These stages have been implicated in the epidemiology of breast cancer risk^8^,^9^, but how they may impact DSB repair is unknown.

Reporters containing repeat sequences have been used to detect homologous recombination events in both somatic and germ line cells of the mouse for almost two decades, but typically these events are rare^10^,^11^,^12^,^13^,^14^,^15^. Furthermore, when assaying spontaneous events, variation between animals can be high, requiring the use of large numbers of mice for mutant comparisons^14^. Thus, novel systems need to be developed to interrogate HDR within animal tissues.

Here we develop such a system where a defined lesion—a DSB—is efficiently introduced into the genome, allowing us to examine HDR within proliferative tissues. In this system, we couple mice containing the widely used reporter for HDR, direct repeat-green fluorescent protein (DR-GFP)^16^, with those developed to contain doxycycline-inducible I-SceI endonuclease for DSB formation. Because I-SceI endonuclease has a long recognition site (∼18 bp), its expression is not toxic to cells^17^. We show that HDR of the I-SceI-induced DSB is robust in mammary epithelium during the proliferative stages of puberty and pregnancy, comprising one-third or more of total DSB repair events, and is also frequent in the small intestine epithelium. Mutation of *Brca2* reduces HDR to a similar extent in mammary epithelium and other tissues. Further, mutation of *Brca2* impacts HDR similarly in different mammary epithelial cell lineages, consistent with the heterogeneous nature of BRCA2-deficient breast tumours^18^.

## Results

### High HDR in mammary tissue during puberty and pregnancy

**Figure 1:**
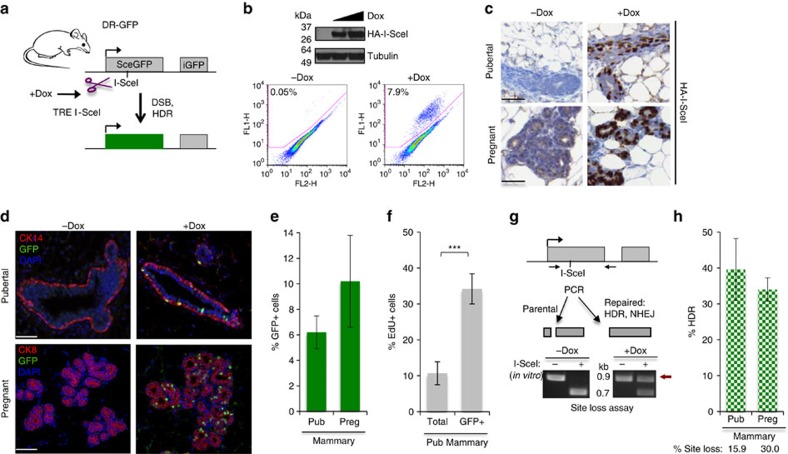
HDR is high in mammary tissue during proliferative stages of development. (**a**) I-SceI DR-GFP mouse model. Dox treatment of mice leads to I-SceI expression. HDR of the I-SceI-induced DSB in *SceGFP* using *iGFP* as template results in GFP expression. (**b**) Primary mammary epithelial cells show high levels of HDR upon Dox addition to the culture. Cells were isolated from an 8-week-old virgin female I-SceI DR-GFP mouse; Dox was added to induce I-SceI expression and 48 h later cells were collected for flow cytometry and western blot analysis. The mean %GFP+ cells is shown. (**c**) I-SceI expression is detected in mammary tissue from pubertal and pregnant mice using an anti-HA antibody. Scale bars, 50 μm. (**d**) HDR is detected by immunofluorescence in mammary tissue from pubertal and pregnant mice using anti-GFP and cytokeratin antibodies (pubertal, CK14; pregnant, CK8). Nuclei are visualized by DAPI. GFP is localized to the nucleus. Scale bars, 50 μm. (**e**) The %GFP+ cells, measuring HDR events, is high in mammary tissue during puberty and pregnancy. Mammary epithelial cells were dissociated from freshly harvested tissue from 6-week-old pubertal mice (*n*=6) and 14.5–17.5 dpc pregnant mice (*n*=9) treated with Dox for 9 days before harvest. Error bars here and in subsequent figures represent s.d. (**f**) The GFP+ population is enriched for EdU+ cells. Glands were harvested from 5-week-old wild-type mice (*n*=4) treated with Dox for 3 days; 24 h before harvest, mice were given a single intraperitoneal injection of EdU at 0.5 mg per10 g mouse weight. ***P=0.0001 (student's *t*-test; two-tailed). (**g**) Site loss assay to measure DSB repair from imprecise NHEJ and HDR combined. The PCR product is cleaved *in vitro* if a DSB was not induced or was precisely repaired *in vivo*, but not cleaved (red arrow) *in vitro* if repair by HDR or NHEJ *in vivo* leads to I-SceI site loss. (**h**) HDR is high in mammary tissue during puberty and pregnancy. The %HDR is the %GFP+ cells determined by flow cytometry divided by % site loss. The % site loss for mice in **e** is shown below.

We previously generated mice containing the HDR reporter DR-GFP integrated into their genome on chromosome 17 (ref. 15) (Fig. 1a). The DR-GFP reporter consists of two defective GFP genes; a DSB introduced into the upstream gene by the I-SceI endonuclease and repaired by HDR with the downstream gene gives rise to GFP+ cells. By contrast, repair by imprecise NHEJ disrupts the DSB site without restoring a functional GFP gene. To study HDR within tissues in the animal, DR-GFP reporter mice were generated that express I-SceI under the control of a doxycycline (Dox)-inducible promoter (Fig. 1a and Supplementary Fig. 1a–d) and driven by CMV-rtTA (ref. 19).

Given the association between HDR gene mutation and mammary tumour predisposition, we first tested this I-SceI DR-GFP system in mammary cells. Epithelial cells were isolated from the fourth inguinal mammary glands from 2–3-month-old virgin mice and Dox was added to the culture for 2 days. Tightly controlled expression of the HA-tagged I-SceI endonuclease was observed in these primary cultures and, concordantly, a large induction in the number of GFP+ cells was detected by flow cytometry (8%; Fig. 1b). The HDR induction is ∼10-fold higher than what we previously observed in primary mammary epithelial cells that were transiently transfected with an I-SceI expression vector^15^, demonstrating the advantage of the inducible system for analysing HDR.

To induce I-SceI within the proliferative mammary epithelium of the animal, pubertal (33–35 days old) and pregnant (5.5–8.5 days post coitum) mice were given Dox in their drinking water for 9 days and the mammary glands were then harvested. Tissue sections stained with an anti-HA antibody demonstrated Dox-dependent expression of I-SceI in both the pubertal and pregnant gland (Fig. 1c). Similarly treated adult virgin mice (2–3 month old) showed many fewer cells expressing I-SceI and so were not further analysed (Supplementary Fig. 1e).

To examine HDR, sections from pubertal mice were stained for GFP as well as cytokeratin 14 (CK14), which is present in the basal cells that make up the outer layer of the duct (Fig. 1d). With I-SceI expression, GFP+ cells were frequent and dispersed throughout the gland in both the luminal and basal layers, demonstrating the non-clonal origin of the cells. By flow cytometry, ∼6% of mammary epithelial cells in the pubertal tissue were GFP+ (Fig. 1e). A similar analysis was performed in mammary tissue during pregnancy, except that cytokeratin 8 (CK8) was used to identify cells lining the lumen (Fig. 1d). As with the pubertal mice, the pregnant mice demonstrated a large number of GFP+ cells dispersed throughout the gland, such that ∼10% of the mammary epithelial cells in the tissue were GFP+ (Fig. 1e). In the absence of Dox, the level of GFP+ cells was substantially lower (pubertal, 0.05%; pregnant, 0.23%; Supplementary Data 1). Thus, with this system HDR can be induced and readily detected within a tissue in the animal.

We next asked whether HDR was enriched in replicating cells. Dox-treated mice were pulsed with EdU for 24 h before mammary gland harvest. Whereas ∼11% of cells in the total epithelial population was GFP+, ∼34% of the EdU-positive cells were GFP+ (Fig. 1f and Supplementary Fig. 1f). These results are consistent with HDR occurring within the S and G2 phases of cycling cells.

NHEJ is often considered the predominant DSB repair pathway in mammalian cells^3^,^4^. To determine the relative contribution of HDR, a PCR-based I-SceI site loss assay was utilized to measure total DSB repair from imprecise NHEJ and HDR^20^ (Fig. 1g). Genomic DNA was collected from cells at the time of GFP analysis for PCR with primers that flanked the break site. The resultant amplification product was cleaved by I-SceI *in vitro* and the fraction of uncleaved DNA (‘site loss', red arrow) arising from DSB repair was determined. Site loss was 16 and 30% in the mouse pubertal and pregnant epithelium, respectively (Fig. 1h), indicating efficient DSB induction and repair. Overall, HDR, as determined by the per cent GFP+ cells relative to site loss for each sample, was observed to be high, such that 40% and 34% of the DSB repair events in the pubertal and pregnant glands were from HDR, respectively (Fig. 1h and Table 1). Thus, in the proliferating mammary gland, HDR is a robust DSB repair pathway.

### HDR varies within different mammary epithelial cell subtypes

**Figure 2:**
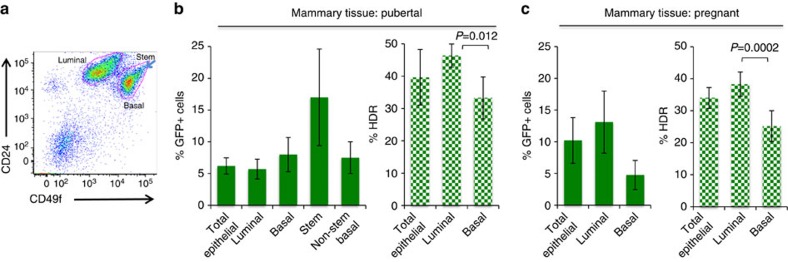
Dependency on HDR varies in different mammary epithelial cell subpopulations. (**a**) Representative flow cytometry dot plot of freshly dissociated mammary epithelial cells stained with anti-CD24 and anti-CD49f antibodies to distinguish luminal (CD24^+^CD49f^low^) and basal (CD24^+^CD49f^high^) populations. Arrow points to putative stem cell subpopulation (top 5% of the basal population). (**b**) Luminal and basal mammary epithelial cells were sorted from wild-type pubertal mice (Fig. 1e,h) and analysed for GFP expression (*n*=6 mice) and HDR (*n*=4 mice), as indicated. The %GFP+ cells in the stem and non-stem cell basal populations are shown. (**c**) Luminal and basal mammary epithelial cells were sorted from wild-type pregnant mice (Fig. 1e,h) and analysed for GFP expression (*n*=9 mice) and HDR (*n*≥6 mice), as indicated.

Mammary epithelium is composed of cells from different lineages including luminal cells that line the duct and the surrounding contractile basal (myoepithelial) cells^21^. Understanding DNA repair in the specific cell populations in the mammary epithelial cell hierarchy is critical to understanding potential effects of cancer-predisposing genetic mutations in the mammary gland, as different lineages may contribute to tumours with specific genetic backgrounds. To examine HDR within different lineages in the mammary gland, epithelial cells dissociated from freshly harvested tissue from Dox-treated mice were co-stained with antibodies to the cell surface markers CD24 and CD49f (ref. 22) (Fig. 2a). In pubertal mice, GFP+ cells were robustly detected in both populations, with the per cent HDR higher in the luminal population, approaching 50% when site loss was taken into account (1.4-fold; Fig. 2b). Similarly, the luminal population during mid-pregnancy was enriched in GFP+ cells and was also confirmed to have a higher level of HDR than the basal population (1.5-fold; Fig. 2c and Table 1).

Mammary stem cells with the hallmark features of multi-lineage differentiation and self-renewal drive growth associated with ductal elongation during puberty in the terminal end buds and are believed to fractionate in the 5% of basal cells that express the highest levels of CD24 and CD49f (refs 23, 24) (Fig. 2a and Supplementary Fig. 1g). Notably, we found that the highest percentage of GFP+ cells in the mammary gland was in this stem cell fraction, ∼2.2-fold over the non-stem cell basal population (Fig. 2b). While it is generally thought that most adult stem cells are slow dividing and exist in a quiescent state, activated stem cells have been proposed to drive proliferative changes during puberty^7^,^25^. Although stem cells represent a small fraction of the basal compartment, a precise repair pathway would be particularly important for these repopulating cells to faithfully transmit genetic information.

### Reduced HDR in mammary tissue from *Brca2* mutant mice

The breast cancer suppressor BRCA2 has been established to play a critical role in HDR in embryonic cells and transformed cell lines^26^. To determine whether *Brca2* mutation differentially affects HDR in mammary epithelium compared with other tissues, we used a hypomorphic allele of *Brca2*, which unlike null alleles of *Brca2*, permits survival of the mice through embryogenesis. The *Brca2*^*Δ27*^allele encodes a peptide that retains binding to several HDR proteins, but lacks the C terminus of the protein that includes a RAD51 interaction site^5^,^27^. A similar frameshift mutation has been found in multiple Dutch families with breast and ovarian cancer^28^. *Brca2*^*Δ27/Δ27*^ mice are viable and fertile, but show an increased incidence of tumours at long latency compared with wild-type littermates, including mammary^27^.

**Figure 3:**
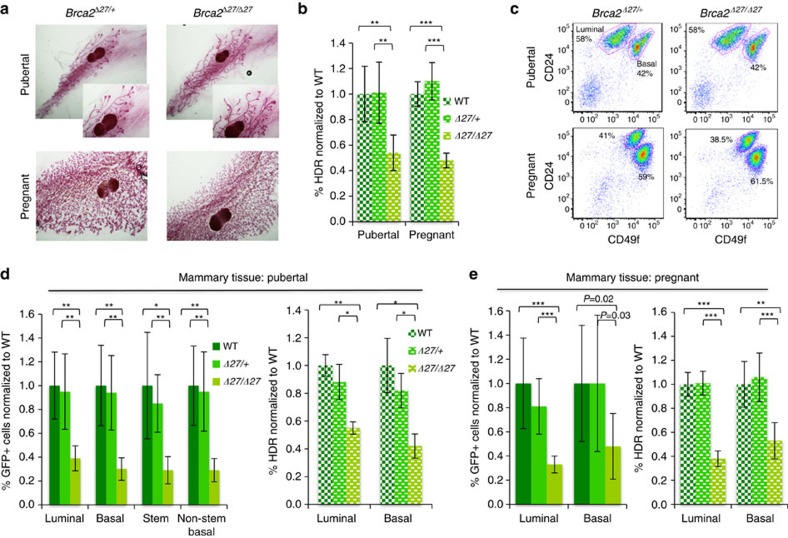
HDR is reduced in mammary epithelium from *Brca2*^*Δ27/Δ27*^ mice. (**a**) Representative whole-mount images of carmine alum-stained fourth inguinal mammary glands from 5-week-old *Brca2*^*Δ27/+*^ and *Brca2*^*Δ27/Δ27*^ littermates (top) and pregnant (dpc 15.5) *Brca2*^*Δ27/+*^ and *Brca2*^*Δ27/Δ27*^ littermates (bottom). (**b**) HDR in mammary tissue is reduced by *Brca2*^*Δ27*^ mutation. The %HDR for *Brca2*^*Δ27/+*^ and *Brca2*^*Δ27/Δ2*7^ mammary tissue is shown normalized to WT for pubertal (*n*≥6 mice of each genotype) and pregnant (*n*≥7 mice of each genotype) mice. Mammary epithelial cells were dissociated from freshly harvested tissue from 6-week-old pubertal mice and 14.5–17.5 dpc pregnant mice treated with Dox for 9 days before harvest; **P≤0.001, ***P≤0.0001 (student's *t*-test; two-tailed). (**c**) Representative flow cytometry dot plots of luminal (CD24+CD49f^low^) and basal (CD24+CD49f^high^) populations from pubertal (6-week old) and pregnant *Brca2*^*Δ27/Δ27*^ (14.5 dpc) mice versus *Brca2*^*Δ27/+*^ controls. Values shown for basal and luminal populations are the percentages of the total number of epithelial cells. (**d**) HDR is reduced in different mammary epithelial cell subpopulations in pubertal *Brca2*^*Δ27/Δ27*^ mice. Mammary epithelial cells of the indicated populations from pubertal mice (Fig. 3b) were analysed for GFP expression (left). Luminal and basal mammary epithelial cells were sorted and %HDR for *Brca2*^*Δ27/+*^ and *Brca2*^*Δ27/Δ2*7^ is shown normalized to WT (*n*≥3 mice of each genotype) for each population (right). *P≤0.01, **P≤0.001 (student's *t*-test; two-tailed). (**e**) HDR is reduced in different mammary epithelial cell subpopulations in pregnant *Brca2*^*Δ27/Δ27*^ mice. Luminal and basal mammary epithelial cells were sorted from pregnant mice (Fig. 3b) and analysed for GFP expression and HDR, as indicated. Values for *Brca2*^*Δ27/+*^ and *Brca2*^*Δ27/Δ2*7^ are shown normalized to WT for each population (*n*≥6 mice of each genotype). **P≤0.001, ***P≤0.0001 (student's *t*-test; two-tailed). The legends for the two panels are the same as the two panels shown in **d**.

Pubertal *Brca2*^*Δ27/Δ27*^ mice showed no significant alteration in ductal morphology compared with heterozygous littermates (Fig. 3a). Similarly, expansion of the alveolar tree was normal during pregnancy, and *Brca2*^*Δ27/Δ27*^ mice were able to nurse their pups. Mammary tissue from *Brca2*^*Δ27/Δ27*^ and *Brca2*^*Δ27/+*^littermates showed similar staining for the proliferation marker Ki67 (Supplementary Fig. 2a), and the number of S phase cells was similar when mice were pulsed with the thymidine analogue EdU (Supplementary Fig. 2b).

To analyse HDR in the mammary tissue of *Brca2* mutant mice, we used the same Dox course described above for wild-type animals. I-SceI expression was similar in mutant and control littermates (Supplementary Fig. 2c), and comparable levels of I-SceI site loss were also observed (Supplementary Fig. 2d and Table 1). The number of GFP+ cells was found to be significantly reduced in pubertal and pregnant mice with *Brca2* mutation compared with wild-type or *Brca2*^*Δ27/+*^mice; taking into account I-SceI site loss, the reduction in the per cent HDR was approximately twofold in the mutant animals. (Fig. 3b, Supplementary Fig. 2e and Table 1). Thus, *Brca2* truncation at the C terminus led to a clear defect in HDR in mammary tissue.

### *Brca2* ^*Δ27*^ mutation reduces HDR in different mammary lineages

We also examined HDR in the different lineages of the mammary gland of the *Brca2* mutant mice. The lineage composition of the gland was unaffected in the mutants during puberty or pregnancy (Fig. 3c). During puberty, *Brca2*^*Δ27/Δ27*^ mice showed a similar reduction in the per cent GFP+ cells in the luminal and basal populations, resulting in an overall ∼2–2.5-fold reduction in HDR (Fig. 3d and Supplementary Fig. 2f). As in control mice, the stem cell population from *Brca2*^*Δ27/Δ27*^ mice was enriched for GFP+ cells, but the mutant stem cells showed a similar reduction compared with non-stem basal or luminal cells. During pregnancy, the percentage of GFP+ cells in the luminal or basal populations from *Brca2*^*Δ27/Δ27*^ mice was also reduced compared with control mice, although to a somewhat larger extent in the luminal compared with basal population (HDR: 2.6-fold versus ∼2-fold, respectively; Fig. 3e and Supplementary Fig. 2g).

### Similar HDR defects in other tissues from *Brca2* ^*Δ27/Δ27*^ mice

**Figure 4:**
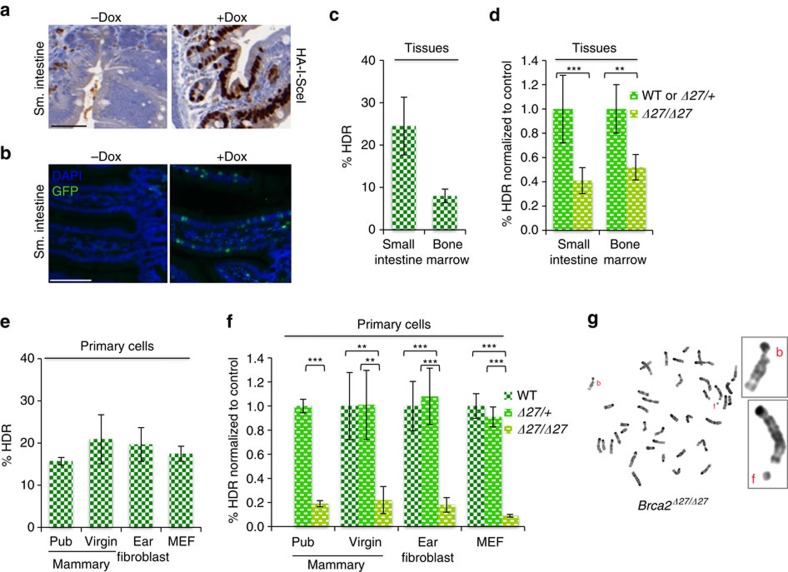
*Brca2*^*Δ27*^ mutation reduces HDR similarly in other tissues and different primary cell types. (**a**) Immunohistochemistry staining of small intestine tissue sections with an anti-HA antibody demonstrating Dox-dependent I-SceI expression. Staining in the absence of Dox is due to antibody trapping since I-SceI is a nuclear protein. Scale bar, 50 μm. (**b**) Representative immunofluorescence image demonstrating HDR events after Dox treatment in small intestine epithelium using an anti-GFP antibody. Nuclei are visualized by DAPI. Scale bar, 100 μm. (**c**) HDR in other proliferative tissues. Small intestinal epithelial cells were harvested from 6 to 9-week-old male and female Brca2^Δ27/+^ mice (*n*=8) following a 9 day course of Dox. Total bone marrow was harvested from 6-week-old female Brca2^+/+^and Brca2^Δ27/+^ mice (*n*=7). (**d**) HDR is reduced in other tissues in *Brca2*^*Δ27/Δ27*^ mice. The %HDR in small intestine epithelium (*n*=8) and total bone marrow (*n*=5) from *Brca2*^*Δ27/Δ2*7^ mice is shown normalized to controls. **P≤0.001, ***P≤0.0001 (student's *t*-test; two-tailed). (**e**) HDR is similar in mammary and other primary cell types in culture. %HDR is shown for primary mammary epithelial cells from 5-week-old (pubertal) *Brca2*^*Δ27/+*^ mice (*n*=3), primary mammary epithelial cells (*n*=6 mice) and ear fibroblasts (*n*=7 mice) from 8 to 12-week-old adult virgin wild-type mice and primary embryonic fibroblasts from E14.5 wild-type embryos (*n*=5 embryos). (**f**) *Brca2*^*Δ27*^ mutation substantially reduces HDR in primary mammary epithelial cells and other cell types. The %HDR in primary mammary epithelial cells (*n*≥4 mice) and ear fibroblasts (*n*≥7 mice) from adult virgin *Brca2*^*Δ27/+*^ and *Brca2*^*Δ27/Δ2*7^ mice and MEFs (*n*≥3 embryos) from E14.5 *Brca2*^*Δ27/+*^ and *Brca2*^*Δ27/Δ2*7^ embryos is shown normalized to WT for each cell type. The %HDR in primary mammary epithelial cells (*n*=3 mice) from *Brca2*^*Δ27/Δ2*7^ pubertal mice is shown normalized to *Brca2*^*Δ27/+*^controls. See Supplementary Data 1 for specific n for each genotype. **P≤0.001, ***P≤0.0001 (student's *t*-test; two-tailed). (**g**) Metaphase spread with representative aberrations (insets) from primary mammary epithelial cells derived from *Brca2*^*Δ27/Δ2*7^ mice showing a chromatid break (**b**) and a chromosome fragment (**f**) adjacent to a normal chromosome.

For comparison with mammary epithelium, we analysed HDR in another highly proliferative tissue, the small intestine. Like the pubertal and pregnant mammary gland, a high percentage of cells in the small intestine are proliferating, as seen by Ki67 staining (Supplementary Fig. 3a) and EdU incorporation (∼12%; Supplementary Fig. 3b). GFP expression is nearly ubiquitous in small intestine cells of mice that constitutionally have a recombined DR-GFP locus (92%; Supplementary Fig. 3c), as with mammary gland^15^, indicating that GFP expression in these two tissues is comparable once HDR has occurred. I-SceI was readily detected in the small intestine epithelium of I-SceI DR-GFP mice treated with the same 9-day course of Dox (Fig. 4a).

GFP+ cells were observed dispersed throughout the epithelium with Dox treatment (Fig. 4b). To quantify HDR, epithelial sheets were isolated and then dissociated into single cell suspensions for flow cytometry. A slightly lower fraction of GFP+ cells was observed in the intestinal epithelial cells compared with the mammary gland but was still substantial (5%; Supplementary Fig. 3d and Table 1). Considering the observed I-SceI site loss (21%), the overall level of HDR was estimated to be 24.5% (Fig. 4c). We also examined the small intestine epithelium of *Brca2*^*Δ27/Δ27*^ mice. HDR was reduced 2.5-fold relative to *Brca2*^*Δ27/+*^ mice (Fig. 4d), similar to that seen in mammary epithelium of both pubertal and pregnant mice.

HDR in total bone marrow was also analysed. Fewer I-SceI-expressing cells were observed in bone marrow (Supplementary Fig. 3e). Taking into account the lower expression and site loss (∼11%), the overall per cent HDR in control animals was only∼8% (Fig. 4c and Supplementary Fig. 3f). However, in *Brca2*^*Δ27/Δ27*^ animals, bone marrow showed a similar reduction in HDR as in the mammary and small intestine epithelium (Fig. 4d). Thus, *Brca2* mutation does not lead to a mammary tissue-specific defect in HDR but rather diminishes HDR in multiple tissue types, including mammary, hematologic and intestinal tissues.

### *Brca2* ^*Δ27*^ mutation substantially reduces HDR in primary cells

In addition to tissues within the animal, we also examined HDR in primary cells to determine how well results from cultured cells would correlate with those in the animal. Following Dox treatment, I-SceI site loss was extremely high at ∼40% in mammary epithelial and fibroblast cultures, and substantial numbers of GFP+ cells were observed (∼8–10%, Supplementary Fig. 4a). Overall, HDR accounted for ∼20% of repair events in all cell types, including primary mammary epithelial cells from pubertal and adult virgin mice (Fig. 4e). This is significantly lower than that observed in the pubertal mammary tissue itself, illustrating that culture conditions do not recapitulate the normal tissue milieu.

Primary mammary epithelial cells derived from *Brca2*^*Δ27/Δ27*^ pubertal or adult virgin mice and ear fibroblasts were found to have an approximately fivefold reduction in HDR; primary embryonic fibroblasts (MEFs) showed an even larger reduction (11-fold; Fig. 4f and Supplementary Fig. 4a), as they did when I-SceI was expressed from a transfected vector rather than induced with Dox (Supplementary Fig. 4b). Not surprisingly, given the substantial HDR defect, the primary *Brca2*^*Δ27/Δ27*^ mammary cells presented with a high number of chromosome aberrations, especially chromatid breaks (Fig. 4g and Supplementary Fig. 4c). The S phase population was found to be reduced only ∼20% in the *Brca2*^*Δ27/Δ27*^ cells compared with controls (Supplementary Fig. 4d), which alone seems unlikely to account for the much greater HDR defect seen in culture, although it suggests that the mutant cells are under stress, even at very low passage. Thus, a similar HDR defect is observed with *Brca2* mutation in both mammary and fibroblast cultures, consistent with the lack of a mammary cell-specific effect. Interestingly, the defect observed in culture is larger than that observed in the animal, even for cells derived from the same tissue (that is, pubertal mammary epithelial cells had fivefold reduced HDR in culture compared with twofold within the gland). Whether the normal tissue environment minimizes the effect of HDR gene mutation or the abnormal culture condition exacerbates HDR defects is not clear.

## Discussion

The importance of HDR in the animal has been unclear. Prior estimates have suggested low levels of HDR in tissues^29^, or were inferred from indirect measures, such as toxicity after exposure to DNA damaging agents and DNA damage-induced foci formation^30^,^31^. The efficient induction of a DNA lesion at a defined genomic location is a powerful approach to understanding DNA repair, and specifically HDR, within the organism. HDR is expected to be highest when cells are cycling, and although many adult tissues are not highly proliferative, cells do proliferate during developmental periods and for self-renewal and wound healing. In particular, the mammary gland undergoes significant proliferative changes after birth and is at risk for tumour development when HDR genes are mutated^6^.

To address the contribution of HDR, we have developed an approach to interrogate DNA repair within the organism by inducing a DSB at a defined genomic location within the context of an HDR reporter. With this approach, DSB induction in proliferating mammary tissue is efficient as is repair by HDR. In keeping with the cell cycle preference for HDR, events are enriched in S/G2 phase cells. A large fraction of detected DSB repair events in pubertal or pregnant mammary epithelium arise from HDR (34–40%), providing quantitative evidence that HDR is a major DSB repair pathway in this tissue.

A significant advantage of our mouse model, with particular relevance to the mammary gland, is the ability to identify specific epithelial cell populations that have undergone HDR, since different cell lineages may have different DNA repair capacities which could impact tissue development and tumour predisposition. During puberty, HDR is greater in the luminal population than in the basal population, in keeping with its larger fraction of proliferative cells^32^. Etiologically, luminal cells contribute to the predominant types of human breast tumours^33^. Notably, however, the highest levels of GFP+ cells in the pubertal gland are in the putative stem cells, a small population identified based on cell surface marker expression. During pregnancy, a significantly larger number of GFP+ cells were observed in the luminal population, such that HDR was ∼1.5-fold greater than in the basal population. Studies have suggested the existence of pregnancy-specific multipotent progenitors that may control alveolar expansion^34^,^35^,^36^, and these progenitors are thought to be in the luminal population^34^.

The small intestine epithelium also showed substantial HDR, although not as high as that in the proliferative mammary epithelium. It will be interesting to determine if HDR varies within lineages in the small intestine epithelium. The higher level measured in mammary tissue suggests that the hormone-dependent proliferative stages of mammary gland development may have greater utilization of HDR compared with other tissues, such that HDR gene mutations compromise genomic integrity more in mammary tissue than other tissue types.

BRCA2, together with BRCA1, are the most frequently mutated HDR genes in breast cancers^6^. The mouse *Brca2*^*Δ27*^ hypomorphic mutation models a truncation mutation found in multiple families with breast and ovarian cancer^28^. Mammary tissue during puberty and pregnancy from these mice have a similar two–three-fold HDR defect as in other proliferative tissue types, including small intestine epithelium, suggesting that an HDR defect *per se* may not confer tissue specificity for BRCA2-associated breast cancers. Our results instead suggest that a greater reliance on HDR to repair DSBs may magnify the effect of *BRCA2* deficiency, making the mammary epithelium more predisposed to tumorigenesis. Whether an increased reliance on HDR is due to cycles of proliferation leading to replication-associated DNA damage and possibly other sources of DNA damage, as from estrogen^37^,^38^, is unclear.

It is notable that while breast and ovarian cancers are particularly prevalent, *BRCA2* mutation carriers are predisposed at lower penetrance to a range of cancers, including pancreas and prostate^5^. Further, young children with biallelic *BRCA2* mutations are at high risk for several tumour types including of the brain, which is still undergoing significant levels of proliferation during childhood^39^. Thus, the requirement for BRCA2 in HDR in other tissues is consistent with *BRCA2* mutations impacting tumour predisposition in multiple tissue types. Clearly, however, other factors besides HDR levels are likely to affect tumour predisposition in any particular cell type; for example, it has been argued that cell turnover can protect the small intestine from tumorigenesis resulting from BRCA2 deficiency^40^.

Although not as dramatic as with pregnancy, the mammary gland undergoes continuing cycles of hormone-driven proliferation during each estrous cycle throughout a woman's reproductive lifespan. Further, proliferation that occurs during pregnancy and each estrous cycle is followed by extensive tissue remodelling to restore the gland to a ground state^41^. Thus, it seems possible that the recurrent, developmental changes may alter the DSB repair-associated effects of *BRCA2* mutation over a lifetime. Even when extensive remodelling occurs, not all cells are likely to be eliminated.

Determining how *BRCA* mutations affect the repair proficiencies of specific mammary epithelial subpopulations is relevant to understanding the cell-of-origin of breast cancers^42^. Since the requirement for BRCA2 for efficient HDR is thought to be central to its tumour suppressor role, our observation that *Brca2*^*Δ27*^ mutation reduced HDR similarly in each of the mammary epithelial cell subpopulations analysed suggests that multiple possible cells-of-origin for mammary tumours are at risk with *BRCA2* mutation, consistent with the heterogeneous nature of BRCA2-associated breast cancers. The finding of high numbers of GFP+ cells in the pubertal mammary stem cell population, although not fully interrogated for HDR contribution by site loss determination, suggests that the genomic instability incurred in these cells as a result of BRCA2 deficiency would be propagated to both luminal and basal daughter cells, which would also contribute to tumour heterogeneity. Given that BRCA1-associated tumours have a more defined cell origin^43^,^44^, it will be interesting to determine whether HDR is affected in specific lineages with BRCA1 deficiency.

## Methods

### Mice

Animal care and all animal experiments were performed with the approval of the Memorial Sloan Kettering Cancer Center (MSKCC) IACUC. Mice were housed in the animal facility at MSKCC in accordance with institutional guidelines with 12-hour light and 12-hour dark cycles. I-SceI expression was induced in mice by administering 3 mg ml^−1^ Dox in drinking water with 5% sucrose. Dox water was replaced every 72 h over a 9-day course. For timed mating, the day of vaginal plug detection was designated 0.5 dpc. CMV-rtTA transgenic mice and the *Pim1*^*DR-GFP*^and *Brca2*^*Δ27*^alleles have been described^15^,^19^,^27^. Mice were maintained on a mixed genetic background.

For genotyping, genomic DNA was extracted from tail tips using the Gentra Puregene kit (Qiagen) following the manufacturer's protocol. PCR reactions were performed with 100 ng genomic DNA and 15 pmole of each primer using PuReTaq PCR beads (GE Healthcare). DR-GFP mice were genotyped using primers Pim1Ex1F 5′-AAGATCAACTCCCTGGCCCACCTGCG-3′; Pim1Ex4R 5′-TGTTCTCGTCCTTGATGTCG-3′ and Hyg3A 5′-CCGCTCGTCTGGCTAAGAT-3′ and the following conditions: 94 **°**C for 3 min; 35 cycles of 94 **°**C for 45 s, 62.9 **°**C for 45 s and 72 **°**C for 1 min and a final extension step at 72 **°**C for 15 min. PCR products (786 bp for wild-type allele and 958 bp for the targeted allele) were separated by electrophoresis on a 1.2% agarose gel. The CMV-rtTA transgene was detected using rtTA-specific forward primer 5′-GCTTGGTGTAGAGCAGCCTACAC-3′ and reverse primer 5′-CAGCGCTGAGTGCATATAACGCG-3′ and the following conditions: 94 °C for 4 min; 35 cycles of 94 °C for 30 s, 55 °C for 30 s and 72 °C for 30 s and a final extension step at 72 °C for 7 min (ref. 19). An ∼310 bp product indicates presence of the transgene. *Brca2*^*Δ27*^ mice were genotyped using primers B2F 5′- GGAGGAGGAGGAGTTGTTGA-3′; B2R1.WT 5′-GTCCGGCAGAGAAACGCCACTG-3′ and B2R2.MUT 5′-CAAAAAAGCCCAGATGATGAG-3′ and the following conditions: 94 **°**C for 2 min; 30 cycles of 94 **°**C for 1 min, 65 **°**C for 1 min and 72 **°**C for 1 min and a final extension step at 72 **°**C for 7 min. PCR products (∼930 bp for wild-type allele and 680 bp for the mutant allele) were separated by electrophoresis on a 1.2% agarose gel.

### Generation of inducible I-SceI mice

A linearized 1.5 kb fragment of DNA containing seven direct repeats of the tet operator sequence and I-SceI-coding sequence excised from pWHE-320-HA-I-SceI (ref. 45) by AvaI digestion was injected into fertilized F2 eggs obtained from mating of (C57BL/6J × CBA/J) F1 mice (Supplementary Fig. 1a). Genomic DNA was extracted from tail tips of 66 pups, digested with BsrGI and analysed for the presence of the TRE-I-SceI transgene by Southern blot analysis using a tet-operon-specific probe (Supplementary Fig. 1b). Three TRE-I-SceI founder lines were identified. Two low-copy TRE-I-SceI founders gave rise to lines with robust, controlled expression of I-SceI (Supplementary Fig. 1c). Line #10 was chosen for use in all subsequent analyses. The insertion site for the TRE-I-SceI transgene in the Founder 10 line was mapped to Chromosome 6 by chromosome walking using the GenomeWalker Universal kit (Clontech), and it was found that the transgene locus could be bred to homozygosity, consistent with its benign genomic location (Supplementary Fig. 1c,d). Founder 10 I-SceI mice were genotyped using primers F10.For.Chr6 5′-CTACATGTGGCAACTGGCTCAATCA-3′; F10.Rev.350 5′-CTGAGCCATCTCTCTAGCCCA-3′ and SCE.Rev 5′-CTGCTTATATAGGCCTCCCACCGTACA-3′ and the following conditions: 95 °C for 3 min; 32 cycles of 95 °C for 30 s, 58.5 °C for 30 s and 72 °C for 1 min and a final extension step at 72 °C for 5 min. PCR products (350 bp for wild-type band and 550 bp for I-SceI) were separated by electrophoresis on a 1.5% agarose gel.

### Mammary epithelial cell purification and flow cytometry

For tissue experiments, all mice were homozygous for the TRE-I-SceI transgene. Fourth inguinal mammary glands were dissected from 6-week-old female mice for puberty, and P14.5-17.5 at 10–13 weeks of age for pregnancy experiments. Lymph nodes were removed, and fresh tissue was minced with a razor blade and incubated for 1 h at 37 °C with gentle shaking in DMEM/F12 containing 2 mg ml^−1^ collagenase (Roche) and a mixture of 33 U ml^−1^ hyaluronidase and 100 U ml^−1^ collagenase (StemCell Technologies, Inc.) with 5 μg ml^−1^ insulin. Organoids were isolated by differential centrifugation and were dissociated in 0.2% trypsin-EDTA for 5 min followed by 5 U ml^−1^ dispase (StemCell Technologies, Inc.) with 0.1 mg ml^−1^ DNase I (StemCell Technologies, Inc.) for 5 min. Cells were filtered through a 40 μm cell strainer, and negative selection against Lin^+^ cells was done using the EasySep Mouse Epithelial Cell Enrichment kit (Stem Cell Technologies). Lin^−^ (CD45^−^ CD31^−^ TER119^−^ BP-1^−^) cells were blocked for 10 min on ice in Hank's balanced salt solution (HBSS) containing 10 mM HEPES and 10% fetal bovine serum (FBS) followed by immunostaining for 10 min on ice in HBSS containing 10 mM HEPES and 2% FBS with PE/Cy7 anti-mouse CD24 (1:400, BioLegend, 101821) and Alexa Fluor 647 anti-human/mouse CD49f (1:100, BD Biosciences, 562473) antibodies. Cells were filtered through a strainer cap and analysed on a BD FACScan or for sorting experiments dead cells were excluded by DAPI staining and cell populations were sorted using a BD FACS ARIA.

### *In vivo* EdU incorporation

For analysis of EdU incorporation in GFP+ cells, mice on a 3-day course of 3 mg ml^−1^ Dox in drinking water received a single intraperitoneal injection of 0.5 mg EdU in PBS per 10 g mouse weight. After 24 h, mice were killed, and mammary epithelial cells were dissociated from freshly harvested tissue. Single cells were fixed with 4% paraformaldehyde (PFA), stained using the Click-iT Plus EdU Alexa Fluor 647 Flow Cytometry assay kit (ThermoFisher Scientific) and analysed on a BD LSRFortessa. For experiments comparing EdU incorporation in mammary epithelial cells versus small intestine epithelial cells, mice received a single intraperitoneal injection of 0.5 mg EdU in PBS per 10 g mouse weight, and were killed after 24 h and analysed as above. For experiments comparing EdU incorporation in mammary epithelial cells from *Brca2* mutant mice versus heterozygous controls, mice received a single intraperitoneal injection of 0.3 mg EdU in PBS per 10 g mouse weight. After 3 h, mice were killed and mammary epithelial cells were dissociated from freshly harvested tissue, fixed with 4% PFA, stained using the Click-iT EdU Alexa Fluor 488 Flow Cytometry assay kit (ThermoFisher Scientific) and analysed on a BD FACScan.

### I-SceI site loss assays

To estimate the efficiency of I-SceI cleavage, cells were collected at the same time as for flow cytometry for DNA extraction using the Puregene Core Kit A (Qiagen). Sequences flanking the I-SceI site were amplified with forward primer 5′-CCCGCCACCTGCCCCATCTGCTA and reverse primer 5′-CCTCTACAAATGTGGTATGGCTGATTATG. PCR products were cleaved *in vitro* with I-SceI (Fermentas) and run on a 1.5% agarose gel. The intensity of the uncleaved (879 bp) and cleaved (663 bp) bands were quantified using a BioRad ChemiDoc System with rolling disk background subtraction. Because the 663 bp band is less intense than the 879 bp band, a 1.325 (879 bp/663 bp) correction factor was used to calculate the site loss fraction (intensity of the 879 bp band divided by (intensity of the 879 bp band plus intensity of the 663 bp band multiplied by 1.325)).

### Histological analysis

The fourth inguinal mammary glands and segments of the small intestine (duodenum, jejunum) were resected and fixed overnight in 4% PFA and stored in 70% ethanol. PFA-fixed tissues were paraffin embedded and 5 μm sections were used for immunohistochemistry with anti-HA (1 μg ml^−1^; Roche, 11867431001) and anti-Ki67 (0.4 μg ml^−1^;Vector, VP-K451) antibodies or immunofluorescence with anti-GFP (2 μg ml^−1^; abcam, ab13970) and anti-CK8 (TROMA-1; 1:100; Developmental Studies Hybridoma Bank) or anti-CK14 (LL002; 1 μg ml^−1^; abcam, ab7800) antibodies.

### Mammary gland whole mounts

The fourth inguinal mammary glands were dissected, spread onto slides and fixed in Carnoy's fixative overnight. Tissues were rehydrated in successive washes of 70, 50, 30 and 10% EtOH then washed in distilled H_2_O and stained overnight in carmine alum solution (Stem Cell Technologies). After staining, mammary glands were washed successively in 70, 95 and 100% EtOH, cleared overnight in xylene and mounted in Permount (Fisher).

### Isolation of small intestine epithelial cells

Epithelial cells were isolated from the small intestine. Briefly, a 2 cm segment of the duodenum was longitudinally cut open and lightly rinsed with PBS to remove residual luminal contents. The tissue was incubated in 5 ml 20 mM EDTA in PBS at 37 °C for 5 min and then shaken. After shaking, the separated muscle layer was removed and the supernatant containing epithelial sheets was centrifuged at 1,000*g* for 1 min. The pelleted epithelial sheet was washed with 10% FBS in PBS and centrifuged again at 1,000*g* for 1 min. The cells were then resuspended in 2.5 ml of 5 U ml^−1^ dispase (StemCell Technologies, Inc.) with 50 μl of DNaseI (StemCell Technologies, Inc.) and incubated at 37 °C for 10 min with intermittent shaking (15 s shaking every 2 min). Completion of single cell segregation was confirmed with a hemocytometer. Cells were then filtered through a 70-μm filter and centrifuged at 200*g* for 5 min. The resulting pellet was resuspended in 1 ml of HBSS containing 10 mM HEPES and 10% FBS and blocked on ice for 10 min. Cells were then pelleted at 500*g* for 5 min at 4 °C and resuspended in HBSS containing 10 mM HEPES and 2% FBS and incubated on ice for 10 min with PE/Cy7 anti-mouse EpCAM antibody (1:200; BioLegend, 118215). After washing one time, cells were resuspended in 1 ml of HBSS containing 10 mM HEPES and 10% FBS, filtered through a cell strainer cap and analysed on a BD FACScan. EpCAM staining was used to check the purity of each intestine cell preparation, which was similar in *Brca2* mutant and control mice (∼80%; Supplementary Data 1).

### Bone marrow harvest

Bone marrow cells were isolated from the femurs of 6–7-week-old female mice. Skin and muscle surrounding the bone was removed and each femur was placed in a PCR tube with a hole in the bottom within a microfuge tube to collect cells following a brief spin. Pelleted cells were treated with red blood cell lysis buffer (Sigma) for 5 min on ice to eliminate red blood cells. After a brief spin, cells were resuspended in PBS, and GFP expression was analysed on a BD FACScan. Note that slightly reduced GFP expression was observed in bone marrow from recombined DR-GFP mice (76%; Supplementary Fig. 3g), compared with mammary and intestinal epithelial cells.

### Primary cell cultures

All mice used in HDR experiments were hemizygous for the DR-GFP reporter and the CMV-rtTA transgene. Mice were typically hemizygous for the TRE-I-SceI transgene, except where indicated. Mammary epithelial cells were isolated from the fourth inguinal mammary glands of 8–12-week-old virgin female mice and plated in DMEM/F12 containing 10% FBS supplemented with 10 μg ml^−1^ insulin and 5ng ml^−1^ epidermal growth factor. On day 1 after harvest, 150,000 cells were plated in a six-well collagen-coated dish and 18 h after plating, Dox was added to cells at a concentration of 1 μg ml^−1^ to induce I-SceI expression. Cells were harvested for flow cytometry and genomic DNA extraction 48 h later.

Primary fibroblasts were obtained from ears of 2–3-month-old freshly killed mice. Ear tissue was clipped at the base of the ear after soaking in 70% ethanol. Tissues were diced in a Petri dish using a sterile razor, placed into 5 ml of 3 mg ml^−1^ collagenase solution in DMEM and incubated for 3 h at 37 °C with agitation and filtered through a 70 μm cell filter. The filtrate was spun at 400*g* for 10 min, and the cell pellet was resuspended in DMEM with 10% FBS, 1% penicillin-streptomycin (Gemini), and 1% MEM nonessential amino acids (Gibco). On day 2 after harvest, 150,000 cells were plated in a six-well dish and 18 h after plating, Dox was added as above. MEFs were derived from 14.5-days embryos (all embryos were homozygous for TRE-I-SceI). Head and liver were removed, and trunks were minced with a sterile razor blade and dissociated in 1 ml of 0.05% trypsin/EDTA at 37 °C for 45 min followed by pipetting up and down several times in 4 ml high-glucose DMEM with 10% FBS and filtering through a 70 μm cell filter. The filtrate was spun at 400*g* for 5 min, and the cell pellet was resuspended in high-glucose DMEM with 10% FBS. On day 1 after harvest, 125,000 cells were plated in a six-well dish and Dox was added as above.

### Chromosome analysis

Primary mammary epithelial cells at passage 1 were treated with 100 ng ml^−1^ KaryoMAX Colcemid (ThermoFisher Scientific) for 1 h before harvest. Following trypsinization, cells were incubated for 15 min in hypotonic solution (0.075 M KCl), fixed in methanol:acetic acid (3:1), and spotted onto slides. For each sample, 100 DAPI-banded metaphases were imaged to assess chromosomal instability in the form of chromatid breaks, exchanges and radial figures, ring chromosomes, di- and tricentrics and acentric fragments. Chromatid breaks were counted as single-break events, tri-radials and quadri-radials as two break events each. Chromosome aberrations (acentric fragments, di- and tricentrics, ring chromosomes and marker chromosomes) were recorded and the breaks required for these rearrangements were not added to the frequency of chromatid breaks.

### Cell cycle analysis

For cell cycle analysis, primary cells were collected 48 h after plating for HDR assays (24 h before fluorescent-activated cell sorting (FACS)) while cells are actively cycling. Cells were washed twice with PBS and pelleted by centrifugation at 250*g* for 5 min. Cells were fixed in ice cold 70% ethanol and kept at −20 °C until staining (2–7 days). At the time of staining, the fixed cells were washed twice in 2 ml PBS and then resuspended in 0.2 ml staining buffer containing 20 μg ml^−1^ propidium iodide (PI) and 0.2 mg ml^−1^ RNaseA with 0.1% Triton X-100 in PBS.

### Western blot analysis

To detect I-SceI expression, cells were collected at the time of FACS analysis (48 h after addition of doxycycline). Cells were lysed for 20 min on ice in TEGN buffer (10 mM Tris (pH 8), 1 mM EDTA, 10% glycerol, 0.5% Nonidet P-40, 400 mM NaCl) with 1 mM DTT in the presence of protease inhibitors (Roche). Lysates were cleared by centrifugation at 10,000*g* for 10 min. Total protein concentration was determined, and 30 μg protein was loaded onto 10% SDS-polyacrylamide gel electrophoresis gels and transferred to nitrocellulose (Bio-Rad). Membranes were blocked for 1 h in 5% milk prepared in PBS with 0.1% Tween-20 and probed overnight at 4 °C with anti-HA (1:1,000 in 5% milk, Covance clone 16B12, MMS-101) or anti-tubulin antibodies (1:10,000 in 5% milk, Sigma clone DM1A, T9026). After washing, membranes were incubated with anti-mouse horseradish peroxidase-linked secondary antibody (1:10,000 in 5% milk) for 1 h. Proteins were visualized with Western Lightning Plus-ECL Enhanced Chemiluminescence (Perkin Elmer). Uncropped images of Western blots are shown in Supplementary Fig. 5.

### Statistical analysis

All data analyses were performed using GraphPad software. Numerical data are shown as the mean±s.d. Significant differences between sample groups were determined using a Student's *t*-test. *P* values of <0.05 are considered statistically significant and are indicated with asterisks as follows: *P≤0.01, **P≤0.001 and ***P≤0.0001.

### Data availability

All relevant data are available from the authors on request. Raw data for all graphs and tables are provided in Supplementary Data 1.

## Additional information

**How to cite this article:** Kass, E. M. *et al*. Robust homology-directed repair within mouse mammary tissue is not specifically affected by *Brca2* mutation. *Nat. Commun.* **7**, 13241 doi: 10.1038/ncomms13241 (2016).

## Supplementary Material

Supplementary Information Supplementary Figures 1-5.

Supplementary Data 1 Raw data for all graphs and tables in figures and supplementary figures.

## Figures and Tables

**Table 1:** HDR in different epithelial cell types.

Tissue	Cell type	%GFP+ cells (*n*)	%Site loss (*n*)	%HDR* (*n*)
*Mammary: wild-type*				
Pubertal	Total epithelial	6.2±1.3 (6)	15.9±2.9 (6)	39.6±8.6 (6)
	Luminal	5.7±1.6 (6)	11.5±3.9 (4)	46.4±3.6 (4)
	Basal	8.0±2.7 (6)	23.2±8.7 (4)	33.3±6.5 (4)
	Stem	17.0±7.6 (6)	ND†	ND
	Non-stem basal	7.5±2.5 (6)	ND	ND
Pregnant	Total epithelial	10.2±3.6 (9)	30±10.6 (9)	34±3.3 (9)
	Luminal	13.1±4.9 (9)	35.7±14.3 (7)	38.3±3.8 (7)
	Basal	4.8±2.3 (9)	19.9±7.9 (6)	25.2±4.8 (6)
				
*Brca2*^*Δ27/+*^				
Pubertal	Total epithelial	6.1±2.1 (10)	14.9±2.5 (9)	39.9±9.5 (9)
	Luminal	5.4±1.8 (9)	13.6±5.6 (4)	41.0±5.9 (4)
	Basal	7.5±2.5 (9)	35.4±12.0 (3)	27.2±4.1 (3)
	Stem	14.5±4.1 (9)	ND	ND
	Non-stem basal	7.1±2.5 (9)	ND	ND
Pregnant	Total epithelial	8.9±2.8 (10)	23.6±6.1 (10)	37.3±5.0 (10)
	Luminal	10.6±3.0 (10)	27.8±9.3 (9)	38.8±3.8 (9)
	Basal	4.8±2.7 (10)	17.1±7.5 (10)	26.7±5.1 (10)
				
*Brca2*^*Δ27/Δ27*^				
Pubertal	Total epithelial	2.6±0.7 (7)	12.3±3.6 (6)	21.4±5.5 (6)
	Luminal	2.2±0.6 (6)	8.2±3.0 (3)	25.5±2.0 (3)
	Basal	2.4±0.8 (6)	19.8±2.3 (3)	14.0±2.9 (3)
	Stem	5.0±1.9 (6)	ND	ND
	Non-stem basal	2.2±0.7 (6)	ND	ND
Pregnant	Total epithelial	3.7±0.9 (8)	23.1±6.7 (7)	16.2±2.0 (7)
	Luminal	4.3±0.9 (8)	30.1±7.8 (8)	14.5±2.5 (8)
	Basal	2.3±1.3 (8)	17.1±4.9 (7)	13.3±3.8 (7)
				
*Small Intestine*				
*Brca2*^*Δ27/+*^	Total epithelial	5.0±2.1 (8)	20.7±7.1 (8)	24.5±6.8 (8)
*Brca2*^*Δ27/Δ27*^	Total epithelial	1.3±0.5 (8)	13.0±2.5 (8)	10.0±2.6 (8)

GFP, green fluorescent protein; HDR, homology-directed repair.

^*^%HDR is the %GFP+ divided by the %Site loss within each individual sample.

^†^ND, not determined due to the small number of cells in the stem cell population.
