# Supplementary material for: Robust homology-directed repair within mouse mammary tissue is not specifically affected by Brca2 mutation
Source: Nat Commun. 2016 Oct 25;7:13241. doi: 10.1038/ncomms13241 (PMC5093336; doi:10.1038/ncomms13241)
Supplement: Supplementary Information — Supplementary Figures 1-5. [file ncomms13241-s1.pdf]

Supplementary Fig. 1

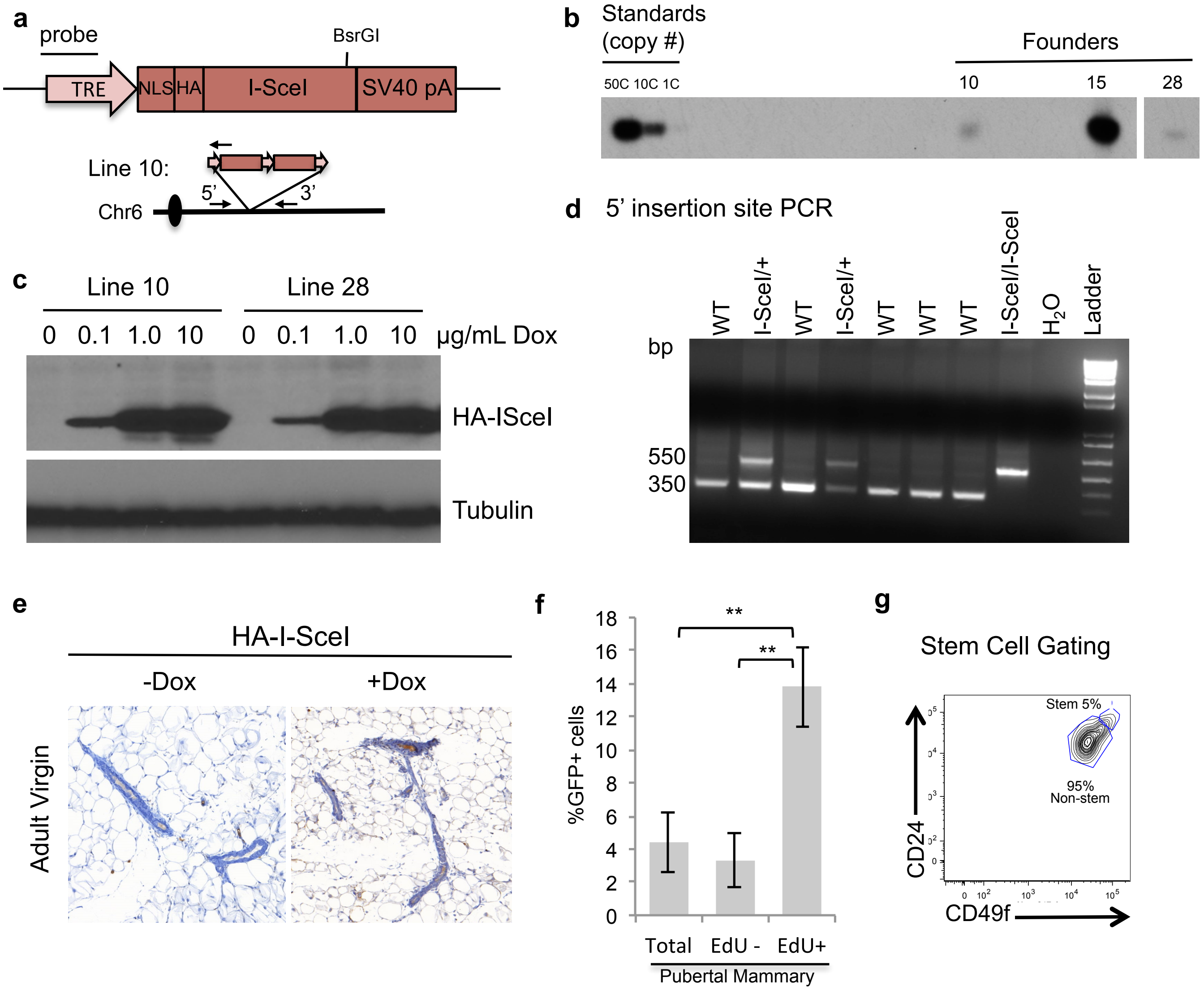

Supplementary Fig. 1. Generation of inducible I-SceI mouse lines

(a) Schematic depicting linearized construct used to generate the TRE-I-SceI mice. Line 10 is estimated to have ~2 direct, tandem copies integrated on chromosome 6 within the Igkv locus, downstream of the Igkv1-131 ORF. Arrows indicate PCR primers used for genotyping.

(b) Southern blot analysis of genomic DNA digested with BsrG1 from three TRE-I-SceI founder lines using the TRE probe shown in (**a**).

(c) Western blot analysis of HA-I-SceI expression levels in ear fibroblasts from mice hemizygous for the TRE-I-SceI (Lines 10 and 28) and CMV-rtTA transgenes treated with increasing concentrations of Dox in culture.

(d) PCR genotyping of Line 10 TRE-I-SceI homozygous, hemizygous and wild-type littermates, using the primers shown in (**a**).

(e) Low levels of I-SceI expression detected in mammary tissue from adult virgin mice using an anti-HA antibody.

(f) The EdU+ population is enriched for GFP+ cells. Glands were harvested from 5-week-old wild-type mice (n=4, mice from Fig. 1f) treated with Dox for 3 days; 24 hours prior to harvest, mice were given a single intraperitoneal injection of EdU at 0.5 mg/10 g mouse weight. **P<0.001 (student's t-test; two-tailed).

(g) Representative flow cytometry contour plot showing gating parameters for distinguishing the stem cell population (top 5% of CD24^{+}CD49f^{high} cells) from non-stem cell basal compartment.

Supplementary Fig. 2

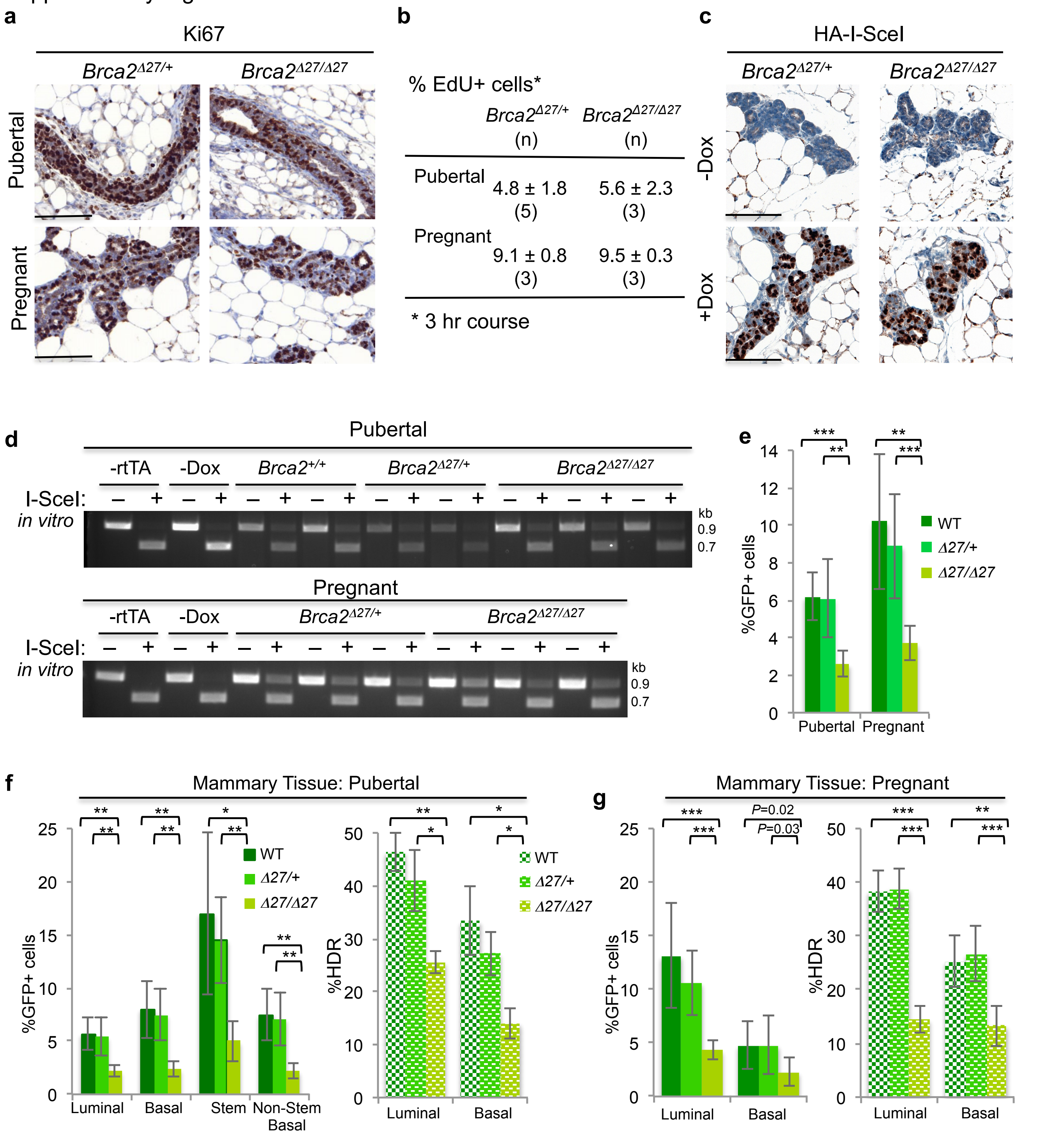

Supplementary Fig. 2. Mammary tissue from *Brca2*$^{Δ27/Δ27}$ mice similar to *Brca2*$^{Δ27/+}$

(a) Immunohistochemistry staining with an anti-Ki67 antibody of mammary tissue sections from 5-week-old *Brca2* $^{Δ27/+}$ and *Brca2*$^{Δ27/Δ27}$ littermates (top) and pregnant (15.5 dpc) *Brca2*$^{Δ27/+}$ and *Brca2*$^{Δ27/Δ27}$ littermates (bottom). Scale bar, 100 microns.

(b) Percent EdU positive cells in freshly dissociated mammary epithelial cells harvested from 5-week-old *Brca2*$^{Δ27/+}$ and *Brca2*$^{Δ27/Δ27}$ mice (top) and pregnant (14.5-15.5 dpc) *Brca2*$^{Δ27/+}$ and *Brca2*$^{Δ27/Δ27}$ mice (bottom), 3 hours after a single intraperitoneal injection of EdU at 0.3 mg per 10 g mouse weight.

(c) Similar I-SceI expression is detected in mammary tissue from pregnant *Brca2*$^{Δ27/+}$ and *Brca2*$^{Δ27/Δ27}$ mice using an anti-HA antibody. Scale bar, 100 microns

(d) Representative site loss gels showing samples from pubertal and pregnant *Brca2*$^{+/+}$, *Brca2*$^{Δ27/+}$ and *Brca2*$^{Δ27/Δ27}$ mice. Genomic DNA was isolated from freshly dissociated mammary epithelial cells at the time of flow cytometry; DNA was amplified using primers flanking the I-SceI site and digested with I-SceI *in vitro*.

(e) The percent of GFP+ cells in mammary tissue from pubertal and pregnant mice is reduced by *Brca2*^{Δ27} mutation (n ≥ 6). Error bars here and in subsequent panels represent standard deviations. $^{**}P≤0.001$, $^{***}P≤0.0001$ (student's *t*-test; two-tailed).

(f) The number of GFP+ cells and percent HDR are similarly reduced in different mammary epithelial cell subpopulations from pubertal *Brca2*$^{Δ27/Δ27}$mice. Absolute numbers for mice in Fig. 3d are shown. $^{*}P≤0.01$, $^{**}P≤0.001$ (student's *t*-test; two-tailed).

(g) The number of GFP+ cells and percent HDR are reduced in luminal and basal populations of mammary epithelial cells from pregnant (14.5-17.5 dpc) *Brca2*$^{Δ27/Δ27}$mice. Absolute numbers for mice in Fig. 3e are shown. $^{**}P≤0.001$, $^{***}P≤0.0001$ (student's *t*-test; two-tailed).

Supplementary Fig. 3

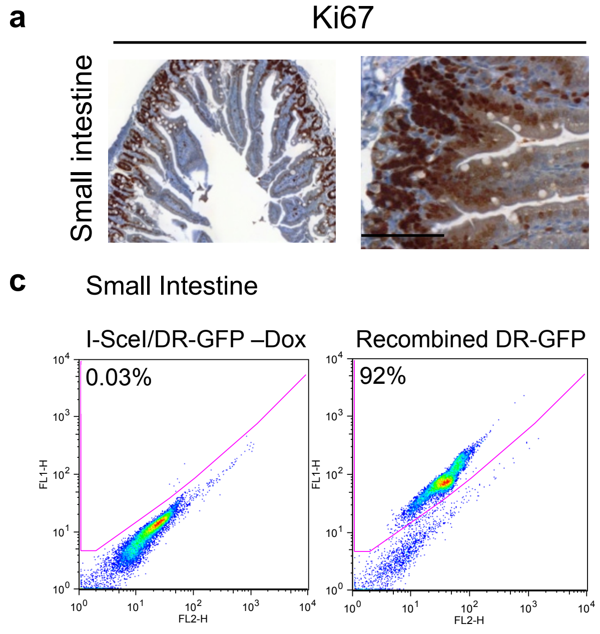

b

Tissue	% EdU+ cells (24h)	n
Mammary Puberty	11.8 ± 5.7	18
Small Intestine	11.5 ± 4.2	12

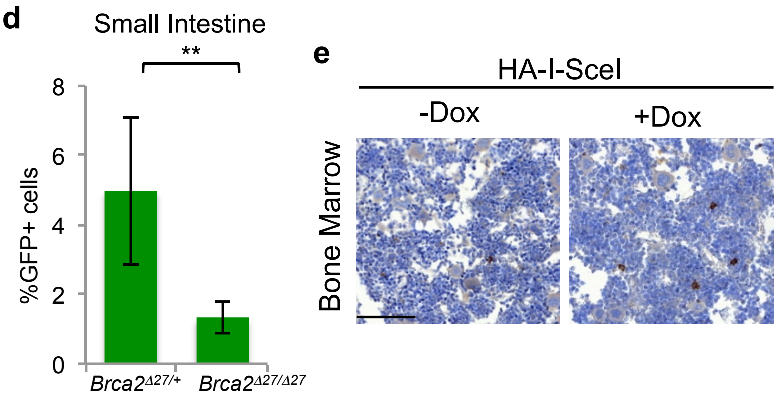

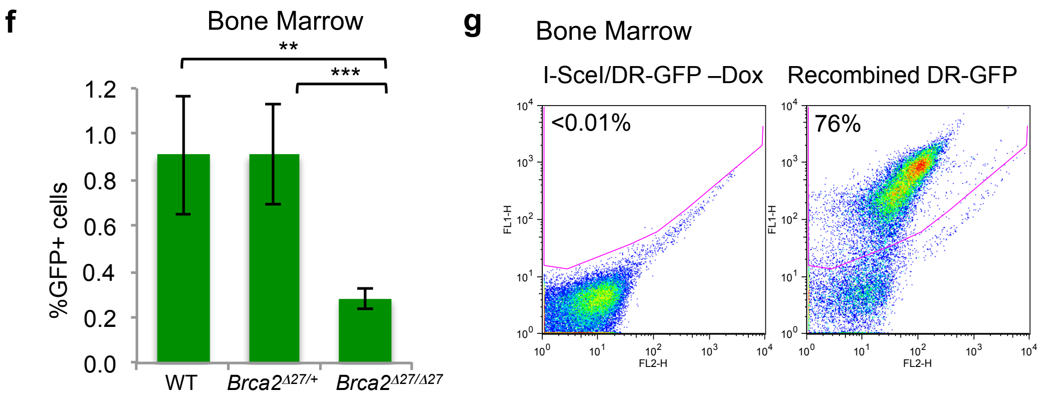

Supplementary Fig. 3. Reduced HDR in proliferative tissues from *Brca2*$^{\Delta 27/\Delta 27}$ mice
(a) Immunohistochemistry staining of small intestine tissue sections with an anti-Ki67 antibody demonstrating areas of high proliferation (right, scale bar is 100 microns).
(b) Percent EdU positive cells in freshly dissociated mammary and small intestine epithelial cells harvested from 5 to 6-week-old wild-type mice 24 hours after a single intraperitoneal injection of EdU at 0.5 mg per 10 g mouse weight.
(c) Representative flow cytometry dot plots of small intestine epithelial cells from a 6-week-old I-SceI DR-GFP mouse (-Dox; left) and a 10-week-old mouse that contains a recombined DR-GFP reporter, resulting in constitutional GFP expression (right).
(d) The number of GFP+ cells is reduced in small intestine epithelium harvested from 6 to 9-week old male and female *Brca2*$^{\Delta 27/\Delta 27}$ mice (n=8) following a 9 day course of Dox, compared to *Brca2*$^{\Delta 27/+}$ controls. Error bars here and in subsequent panels represent standard deviations. $^{**}P \leq 0.001$ (student's *t*-test; two-tailed).
(e) Immunohistochemistry staining of bone marrow from the femur of a 6-week-old female mouse with an anti-HA antibody demonstrating Dox-dependent I-SceI expression. Scale bar, 100 microns.
(f) The number of GFP+ cells is reduced in total bone marrow from *Brca2*$^{\Delta 27/\Delta 27}$ mice compared to controls (n=5). $^{**}P \leq 0.001$, $^{***}P \leq 0.0001$ (student's *t*-test; two-tailed).
(g) Representative flow cytometry dot plots showing %GFP+ cells in total bone marrow from a 6-week-old I-SceI DR-GFP mouse (-Dox; left) and a 6-week-old mouse that contains a recombined DR-GFP reporter, resulting in constitutional GFP expression (right).

Supplementary Fig. 4

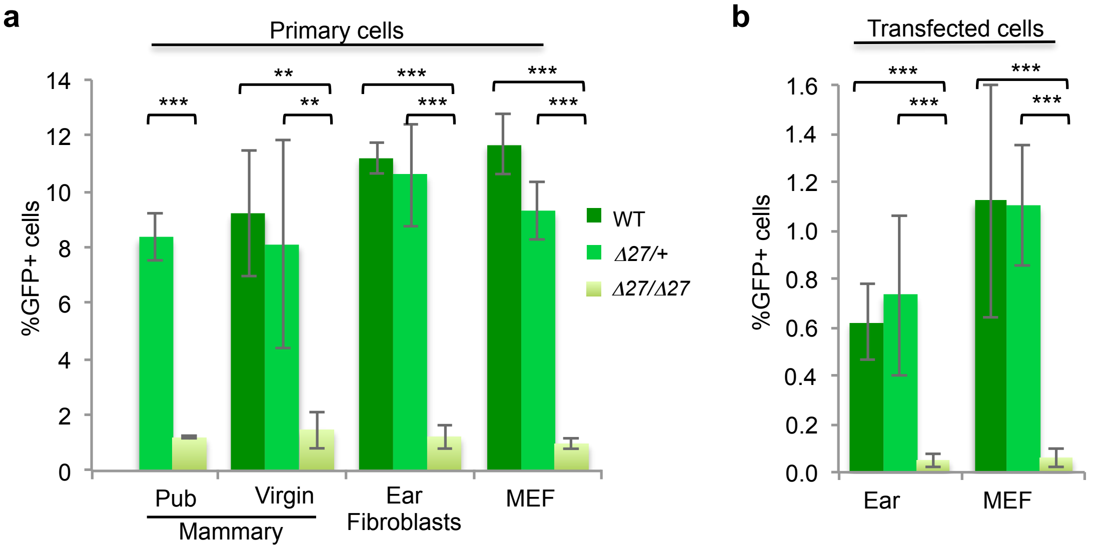

c

Genotype	# of Metaphases	>2n	Metaphases with damage (%)	Total # of Chromo-somes	Fragments	Breaks	Dicentrics/ tricentrics	Markers	(Exchange/ Radial figures)	Total # of events	Avg. Aberrations/ Chromosome (Mean ± S.E.M.)
$Brca2^{\Delta27/+}$	**100**	**4**	**4**	**4161**	**0**	**2**	**0**	**2**	**0**	**4**	**0.001 ± 0.0005**
$Brca2^{\Delta27/\Delta27}$	**100**	**21**	**54**	**4810**	**19**	**62**	**16**	**28**	**8**	**125**	**0.026 ± 0.0038**

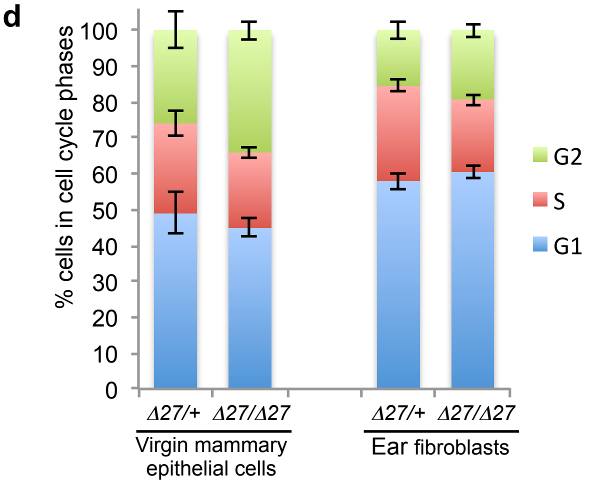

Supplementary Fig. 4. Reduced HDR in primary cells from *$Brca2^{\Delta27/\Delta27}$* mice

(a) Using Dox induction of I-SceI in culture, the number of GFP+ cells is substantially reduced in different primary cell types from *$Brca2^{\Delta27/\Delta27}$* mice. The %GFP+ cells is shown for *$Brca2^{\Delta27/\Delta27}$* primary mammary epithelial cells from pubertal (n=3 mice) and adult virgin mice (n ≥ 4 mice), ear fibroblasts (n ≥ 7 mice) from 8 to 12-week-old mice and MEFs (n ≥ 3 embryos) from E14.5 embryos, compared to *$Brca2^{\Delta27/+}$* and wild-type controls. ***P*≤0.001, ****P*≤0.0001 (student's *t*-test; two-tailed).

(b) Using transient transfection of the I-SceI expression vector, the number of GFP+ cells is reduced in primary ear fibroblasts and MEFs from *$Brca2^{\Delta27/\Delta27}$* mice. Note that the absolute number of GFP+ cells with transient transfection is ~10-fold reduced compared to Dox induction and is more variable. *** p≤0.0001

(c) Chromosome aberrations observed in metaphase spreads from *$Brca2^{\Delta27/\Delta27}$* primary mammary epithelial cells compared to *$Brca2^{\Delta27/+}$* controls. In counting the total number of events, exchanges/radial figures are counted within the break column (two breaks each).

(d) Cell cycle distribution of primary mammary epithelial cells (n ≥3 mice) and primary ear fibroblasts (n ≥5 mice) from *$Brca2^{\Delta27/+}$* and *$Brca2^{\Delta27/\Delta27}$* mice.

Supplementary Fig. 5.

HA (top) and tubulin blots from Fig 1b.

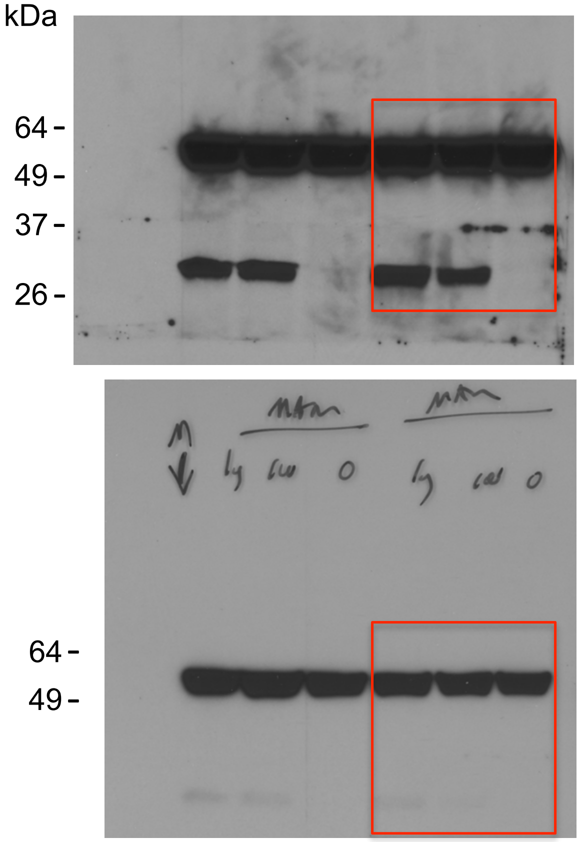

HA (top) and tubulin blots from Supplementary Fig 1c.

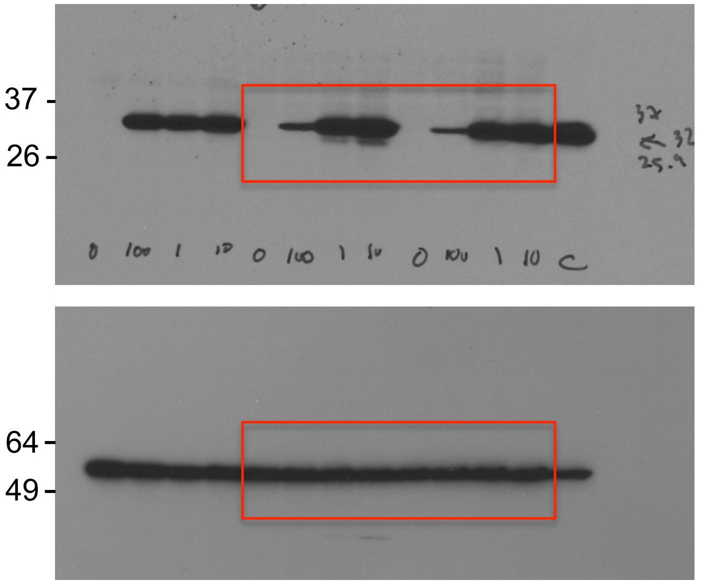

Southern blot from Supplementary Fig. 1b

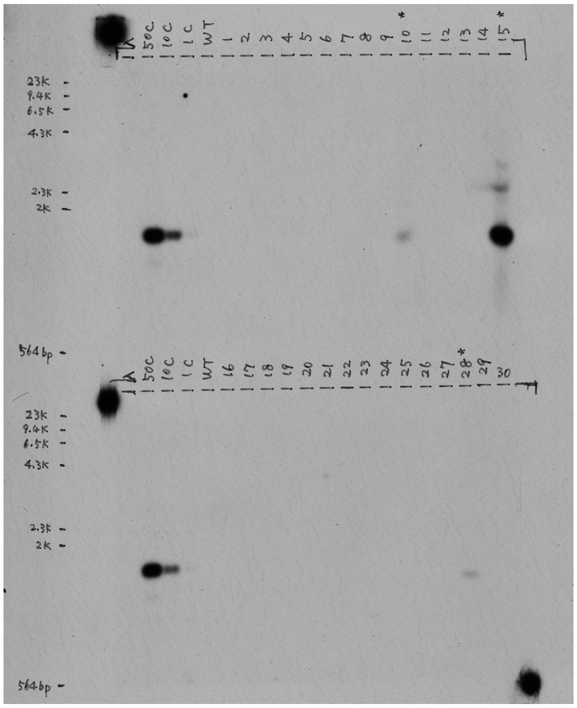

DNA gel from Fig. 1g

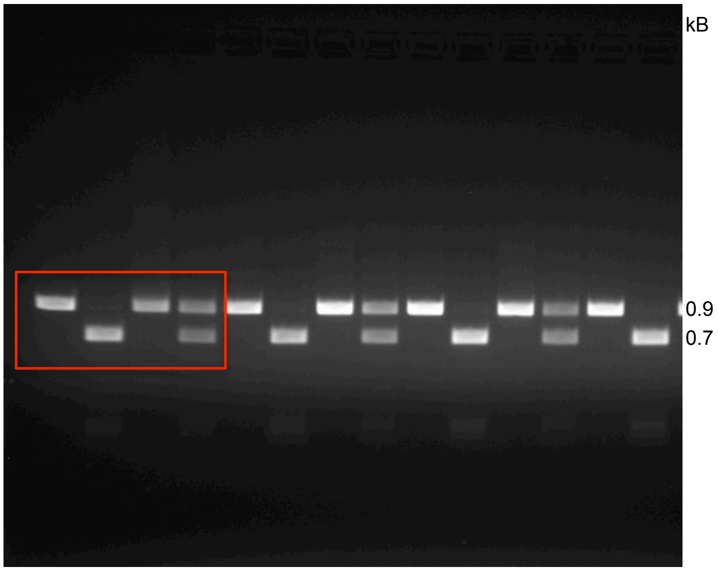

Supplementary Fig. 5.

DNA gels from Supplementary Fig. 2d

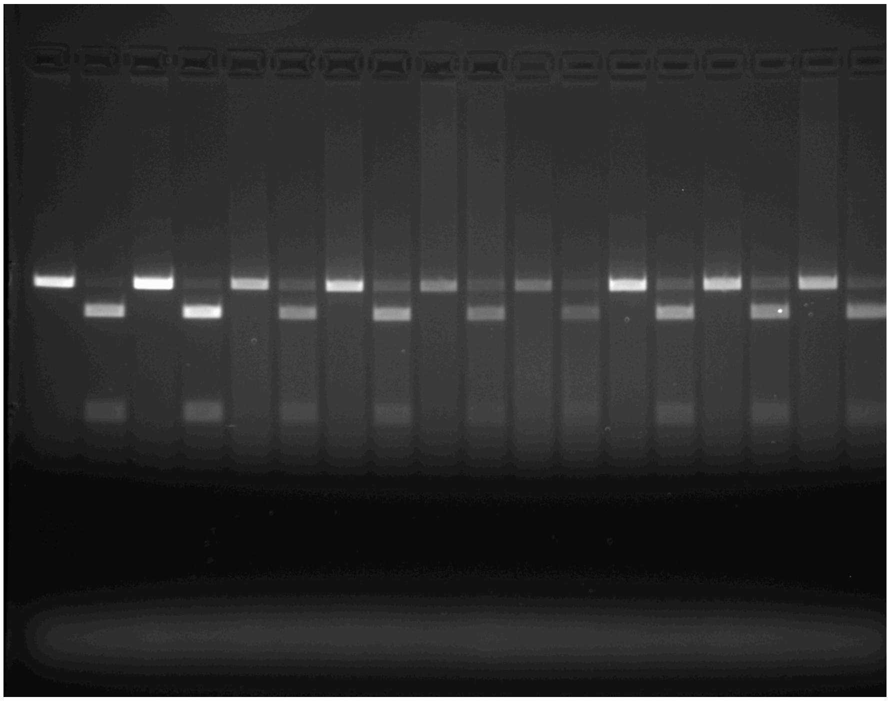

kb

0.9

0.7

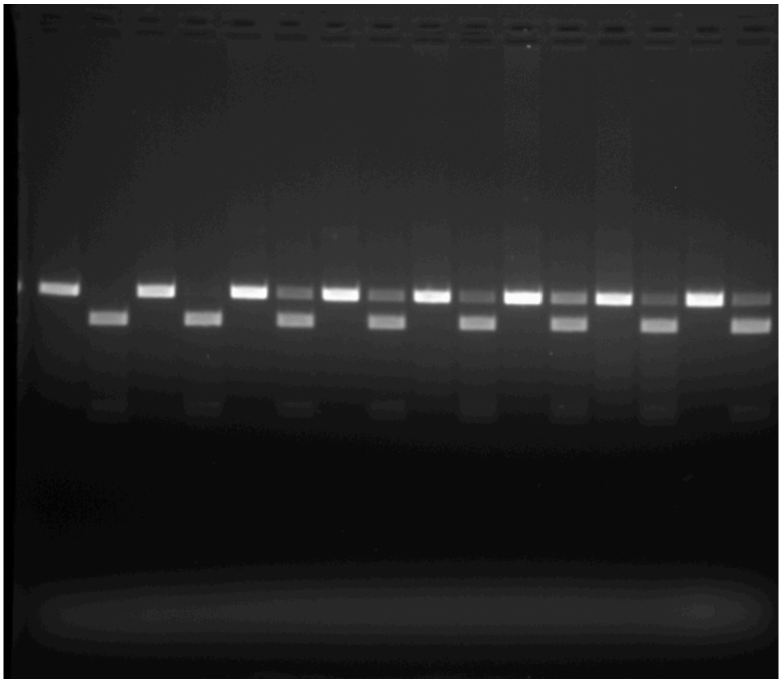

kb

0.9

0.7

Supplementary Fig. 5. Uncropped blots and gels.
